# Leucine-rich repeat kinase-2 (LRRK2) modulates paraquat-induced inflammatory sickness and stress phenotype

**DOI:** 10.1186/s12974-019-1483-7

**Published:** 2019-06-07

**Authors:** Chris Rudyk, Zach Dwyer, Shawn Hayley, CLINT membership, E Schurr, Earl Brown, Derrick Gibbings, Shawn Hayley, David Park, Dana C Philpott, John D Rioux, Michael Schlossmacher

**Affiliations:** Department of Neuroscience, Carleton University, 1125 Colonel By Drive, Ottawa, Ontario K1S 5B6 Canada

**Keywords:** Parkinson’s, LRRK2, Inflammatory, Microglia, Cytokine, Toxicity

## Abstract

**Background:**

Leucine-rich repeat kinase 2 (LRRK2) is a common gene implicated in Parkinson’s disease (PD) and is also thought to be fundamentally involved in numerous immune functions. Thus, we assessed the role of LRRK2 in the context of the effects of the environmental toxicant, paraquat, that has been implicated in PD and is known to affect inflammatory processes.

**Methods:**

Male LRRK2 knockout (KO) and transgenic mice bearing the G2019S LRRK2 mutation (aged 6–8 months) or their littermate controls were exposed to paraquat (two times per week for 3 weeks), and sickness measures, motivational scores, and total home-cage activity levels were assessed. Following sacrifice, western blot and ELISA assays were performed to see whether or not LRRK2 expression would alter processes related to plasticity, immune response processes, or the stress response.

**Results:**

Paraquat-induced signs of sickness, inflammation (elevated IL-6), and peripheral toxicity (e.g., organ weight) were completely prevented by LRRK2 knockout. In fact, LRRK2 knockout dramatically reduced not only signs of illness, but also the motivational (nest building) and home-cage activity deficits induced by paraquat. Although LRRK2 deficiency did not affect the striatal BDNF reduction that was provoked by paraquat, it did blunt the corticosterone elevation induced by paraquat, raising the possibility that LRRK2 may modulate aspects of the HPA stress axis. Accordingly, we found that transgenic mice bearing the G2019S LRRK2 mutation had elevated basal corticosterone, along with diminished hippocampal 5-HT1A levels.

**Conclusion:**

We are the first to show the importance of LRRK2 in the peripheral neurotoxic and stressor-like effects of paraquat. These data are consistent with LRRK2 playing a role in the general inflammatory tone and stressor effects induced by environmental toxicant exposure.

## Background

Virtually, all neurological diseases have an inflammatory component that in some regard is associated with the manifestation of a range of primary and/or co-morbid symptoms. Parkinson’s disease (PD) is no exception having a prominent neuroinflammatory element, along with the range of behavioral and autonomic symptoms, in addition to primary motor deficits [1, 2]. Among the genes implicated in both PD and its inflammatory processes, leucine-rich repeat kinase 2 (LRRK2), and in particular its G2019S mutation, has received considerable attention [3–5]. In fact, the highest levels of LRRK2 were found in peripheral lymphocytes and monocytes of PD patients [6], and LRRK2 mutations have been associated with microglial reactivity and pro-inflammatory cytokine production in the brain and periphery [7–10]. Moreover, typical inflammatory agents, such as lipopolysaccharide (LPS), as well as chemical toxicants like MPTP, that are used to model PD pathology have been shown to influence processes linked to LRRK2 [11–13]. Accordingly, LRRK2 may be a critical general mediator of inflammatory pathology associated with a range of environmental insults.

Numerous reports have implicated environmental toxicants in the development of PD, and in particular, increased incidence of PD has been associated with exposure to various pesticides [14–17]. Indeed, animal studies have confirmed that the herbicide and oxidative stress generating chemical, paraquat, provokes a loss of midbrain dopamine neurons, coupled with a range of behavioral symptoms [18–21]. Strikingly, the impact of pesticide exposure may be modified by genetic background [22–24]. Given the highly variable penetrance of LRRK2 mutations [25, 26], it is likely that environmental stressor exposure of some sort may be required in the context of LRRK2 mutation to provoke a disease state. This is in keeping with the idea that multiple hits give rise to PD; in effect, LRRK2 mutation could “set the stage” or essentially act as the first of such hits over one’s lifetime. Further still, endogenous LRRK2 could underlie the impact of numerous environmental hits by virtue of its role as a modulator of inflammatory immune processes.

The most common LRRK2 mutation linked to PD is the G2019S mutation that is not only associated with the familial form of the disease, but also might act as a risk factor for the idiopathic form of the disease [27, 28]. This mutation is thought to cause a gain of function for the kinase activity of LRRK2, and mice bearing this mutation were reported to show alterations in dopamine levels and enhanced sensitivity to MPTP treatment, as well as increased α-synuclein inclusions [12, 29]. Yet, the literature is mixed with some reports finding no differences in neurodegeneration in G2019S mutants [30]. Interestingly, the G2019S mutation might also increase inflammatory myeloid cell responses to lipopolysaccharide (LPS) [31].

The present focus was on the impact of LRRK2 deficiency and G2019S mutation in response to paraquat. Since paraquat-induced sickness symptoms and behavioral features akin to what is often evident with immune challenges, such as those induced by LPS or TNF-α [32], we sought to evaluate whether LRRK2 knockout (KO) and G2019S transgenic expression would affect the impact of the pesticide on these outcomes. Similarly, it was also of interest to assess whether paraquat provokes alterations in hormonal (corticosterone), neurotransmitter (5-HT1A), trophic (BDNF), and inflammatory (CX3CR1, WAVE2) factors and again whether LRRK2 plays a role. Indeed, we previously found paraquat to reduce BDNF levels [32–34] and a recent report linked LRRK2 mutations to depressive-like symptoms that were correlated with 5-HT1A changes [35]. Members of our group also recently found that the actin regulatory factor, WAVE2, controls macrophage inflammatory response through LRRK2 [36]. Hence, it was expected that the paraquat would not only induce brain changes, but would also have systemic consequences that would involve changes in peripheral inflammatory factors (IL-6) and possibly at the organ level. We presently found that LRRK2 KO prevented motor impairment and signs of peripheral toxicity/stress, including sickness, weight loss, corticosterone, and IL-6 elevations, as well as organ changes in paraquat-treated aged mice. Furthermore, transgenic G2019S overexpression basally altered striatal BDNF, hippocampal 5-HT1A, and corticosterone levels, consistent with a “stress phenotype.” Thus, it appears that LRRK2 is an important regulator of paraquat which provoked peripheral and central effects and may be relevant for neuroimmune toxicity in general.

## Methods

### General study design

Briefly, two parallel studies were conducted on 6–8-month-old LRRK2 KO (JAX# 012444) or BAC transgenic G2019S (JAX #012467) mice along with their respective wild-type (WT) littermate controls that were obtained from our in-house breeding colony. Prior to the commencement of the study, baseline home-cage activity was carried out as described below. Mice were then randomly assigned to one of the four experimental conditions in each of the two studies: study 1 (WT/saline vs WT/paraquat and KO/saline vs KO/paraquat) and study 2 (WT/saline vs WT/paraquat and G2019S/saline vs G2019S/paraquat). These were analyzed as two separate two-way ANOVAs. Saline and paraquat injections (10 mg/kg; 1,1′-dimethyl-4,4-bipyridinium dichloride; Sigma-Aldrich, St. Louis, MO, USA) began 1 day after baseline locomotor measurements and were given every other day for a total of six injections. Motor behavior alterations, assessing home-cage locomotor activity, and fine movement coordination were carried out at various time points as described below. At the end of the experimental paradigm, all animals were rapidly decapitated 1 h following the final injection.

This study used mice that were relatively older (6–8 months) than what we typically use (2–3 months), in order to determine whether LRRK2 is important for the toxic effects of paraquat exposure in slightly older animals. Prior to the main study presented, we initially sought to utilize our dual-hit PD model [37]; however, an initial study revealed profound sickness and mortality in 6–8-month-old mice that received intra-substantia nigra (SNc) infusion of LPS (2 μg) followed 2 days later by nine injections of paraquat (10 mg/kg three times per week). Remarkably, however, this toxicity was greatly diminished in LRRK2 KO mice. Owing to the enhanced mortality, we decided to omit the LPS infusion and curtail the number of injection down to six for the paraquat treatment in these mice in order to explore in detail the role of LRRK2 in pathology. Indeed, the mortality/sickness was typically observed by the sixth injection and this regimen allowed us to ascertain whether LRRK2 KO and/or overexpression of the most common mutation G2019S might impact paraquat toxicity and stressor-like effects.

### Animals

C57BL/6 N-*Lrrk2*^*tm1.1Mjff*^/J KO and B6.Cg-Tg(Lrrk2*G2019S)2Yue/J transgenic mice were purchased from Jackson Laboratories concurrently with C57Bl/6 J mice at 6 weeks of age. All animals were fed Harlan Labs 2018 rodent chow and housed under a normal 12-h light-dark cycle. Males were each mated with 3 females in a harem breeding arrangement until pregnant. At 21 days of age, male pups were ear punched for identification, were placed on Harlan labs 2018 diet and had tail snips taken for genotyping. A total of 48 males from each transgenic line were group housed with littermates and enrichment in individually ventilated cages until 10 weeks of age when they were transferred into an experimental room and group housed until 6–8 months of age at which point they entered the experiment.

For all mice, DNA was extracted from 0.25 cm long tail snips collected at 21 days of age using the Qiagen DNEasy blood and tissue kit. For LRRK2 KO mice, animal genotype was determined via gel electrophoresis following DNA amplification. For G2019S transgenic mice, extracted DNA was analyzed using a BioRad CFX qPCR machine and the recommended Jackson Labs protocol to separate WT animals and heterozygous transgenics based on melt curve data. Pure WT products resulted in melt peaks at 80 °C and heterozygous and homozygous transgenic amplicon products resulted in melt peaks at 84 °C.

### Behavioral analyses

Spontaneous home cage locomotor activity assessment was completed following a 30-min acclimation period in our behavioral testing room, measurements of home-cage locomotor activity occurred once at baseline (day 0), then again the evening of the second and fifth injection.

### Sickness scoring

Sickness symptoms (e.g., ptosis, curled body posture, piloerection) were assessed daily throughout the injection regimen, as previously described [38]. Briefly, animals were assessed for the presence of the following symptoms: ptosis (drooping eyelids), piloerection, curled body posture, and diminished locomotion and/or exploratory behavior. Upon assessment, a score based on the number of symptoms present (0 = no sickness symptoms, 1 = one symptom, 2 = two symptoms, 3 = three symptoms) was applied. Ratings were scored by an observer blind to all experimental conditions.

### Nestlet test

In order to assess goal-directed behavior alterations involving fine motor coordination skills in mice, the nestlet test was used, as previously described [39]. Briefly, 1 day following the second and fifth injection, mice were placed into new standard polypropylene cages (27 × 21 × 14 cm) containing one fully intact nestlet (Ancare, Bellmore, NY) beginning at 08:30. Mice nestlet building behavior was then examined based on the quality of nest built at 1, 3, 5, and 24 h by an investigator blind to all experimental conditions. All scoring was based on the nestlet Likert rating scale whereby scores range from 0 (untouched intact nest) to 6 (perfectly developed nest) [39].

### Home-cage locomotor activity

Spontaneous home cage locomotor activity was measured over a complete 12-h light/dark cycle using our Micromax (MMx) infrared beam-break apparatus (Accuscan Instruments, Columbus, OH, USA), as previously described [33]. Spontaneous home cage locomotor activity assessment was completed following a 30-min acclimation period in our behavioral testing room post nestlet removal, measurements of home-cage locomotor activity occurred once at baseline (day 0), then again the evening of the second and fifth injection.

### Brain dissection and tissue extraction

Half the animals were intraperitoneally administered 200 mg/kg of sodium pentobarbital and perfused with 4% paraformaldehyde. Twenty-four hours later, the brains were transferred to 10% sucrose and then transferred to 30% sucrose 48 h after sacrifice. The remaining animals were sacrificed via rapid decapitation, and trunk blood, brains, and organs were extracted and flash frozen at − 80 °C.

Following rapid decapitation, brains were excised and sectioned into sequential coronal slices using razor blades and a chilled stainless steel microdissecting matrix with adjacent slots spaced ~ 0.5 mm apart. Hollow biopsy needles were then used to collect the dorsal striatum, hippocampus, and SNc. The tissue was immediately frozen upon dissection and was stored at − 80^o^C until processing. Additionally, for the LRRK2 KO study, immediately following sacrifice, the animals’ left lung, spleen, and liver were extracted from the cavity and any excess fat was removed and organs were promptly weighed. The organs were then weight and stored on dry ice and stored at 80^o^C until processing.

### Plasma corticosterone assay

At the time of decapitation, trunk blood from all of the animals was collected in tubes containing 10 μg EDTA. Samples were centrifuged (3000*g* for 8 min), and the plasma removed and stored in aliquots at − 80 °C for later corticosterone determination with commercially available radioimmunoassay kits (ICN Biomedicals, CA, USA). Samples were assayed in duplicate within a single run to control for inter-assay variability; the intra-assay variability was less than 10%.

### Plasma determination of IL-6

Trunk blood was collected at the time of decapitation and prepared as for the corticosterone assay in a separate aliquot at − 80 °C. IL-6 levels were determined using a Luminex Immunoassay (R&D Systems, NE, USA) ran following kit instructions on a Luminex Magpix (Luminex Corporation, TX, USA). Samples were assayed in duplicate within a single run to control for inter-assay variability; the intra-assay variability was less than 10%.

### Western blot

Brain tissue punches and organs were collected to detect levels of BDNF (R&D Systems, MAB248), 5-HT1A (Genetex, GTX104703), CX3CR1 (Sigma-Aldrich, SAB3500204), and WAVE2 (Cell Signaling Technology, 3659), as previously described previously. Briefly, whole cell lysates were homogenized in Radio Immuno Precipitation Assay (RIPA) buffer [50 mM Tris (pH 8.0), 150 mM sodium chloride, 0.1% sodium dodecyl sulphate (SDS), 0.5% sodium deoxycholate, and 1% Triton X-100] mixed with 1 tablet of Complete Mini ethylenediaminetetraacetic acid (EDTA)-free protease inhibitor (Roche Diagnostics, Laval, QC, Cat #11 836 170 001) per 10 mL of buffer. On day 1 of analysis, proteins were separated using sodium dodecyl sulfate-polyacrylamide gel electrophoresis (SDS-PAGE). In order to determine total protein, membranes were incubated in REVERT total protein solution for a period of 5 min followed by placement into a REVERT wash solution (6.7% glacial acetic acid, 30% methanol, in water) two times 2 min each. Membranes were then quickly rinsed with distilled water and imaged on our LI-COR Odyssey imaging system on the 700 channel for an exposure period of 2 min. Membrane incubation with rabbit anti-BDNF, WAVE2, 5-HT1A, and CX3CR1 (1:1000) for a period of 60 min in 0.05% fish gelatin in TBS with 0.1% tween followed by 1 h in infrared anti-rabbit conjugate at a concentration of 1:20,000 in 0.5% fish gelatin solution containing 0.2% tween and 0.01% SDS. Any unbound antibody was removed using 15 mL of TBS-T/membrane, and membranes washed and read on our Licor Odyssey system at the appropriate wavelength for 6 min.

### Statistical analysis

All data was analyzed by 2 (genotype; WT vs. KO or WT vs G2019S) × 2 (injection; saline vs. paraquat) two-way ANOVA with significant interactions further analyzed by means Bonferroni follow-up comparisons (*p* < 0.05) where appropriate. Additionally, analysis of total home cage locomotor activity, sickness scores, and nestlet building behavior was completed using appropriate repeated measures ANOVA’s conducted with time as the third independent variable followed by a post hoc analysis. Data is presented in the form of mean ± standard error mean (mean ± SEM). All data was analyzed using the statistical software StatView (version 6.0), and differences were considered statistically significant when *p* < 0.05.

## Results

### LRRK2 KO prevented paraquat and LPS + paraquat-induced sickness behavior and mortality

**Fig. 1:**
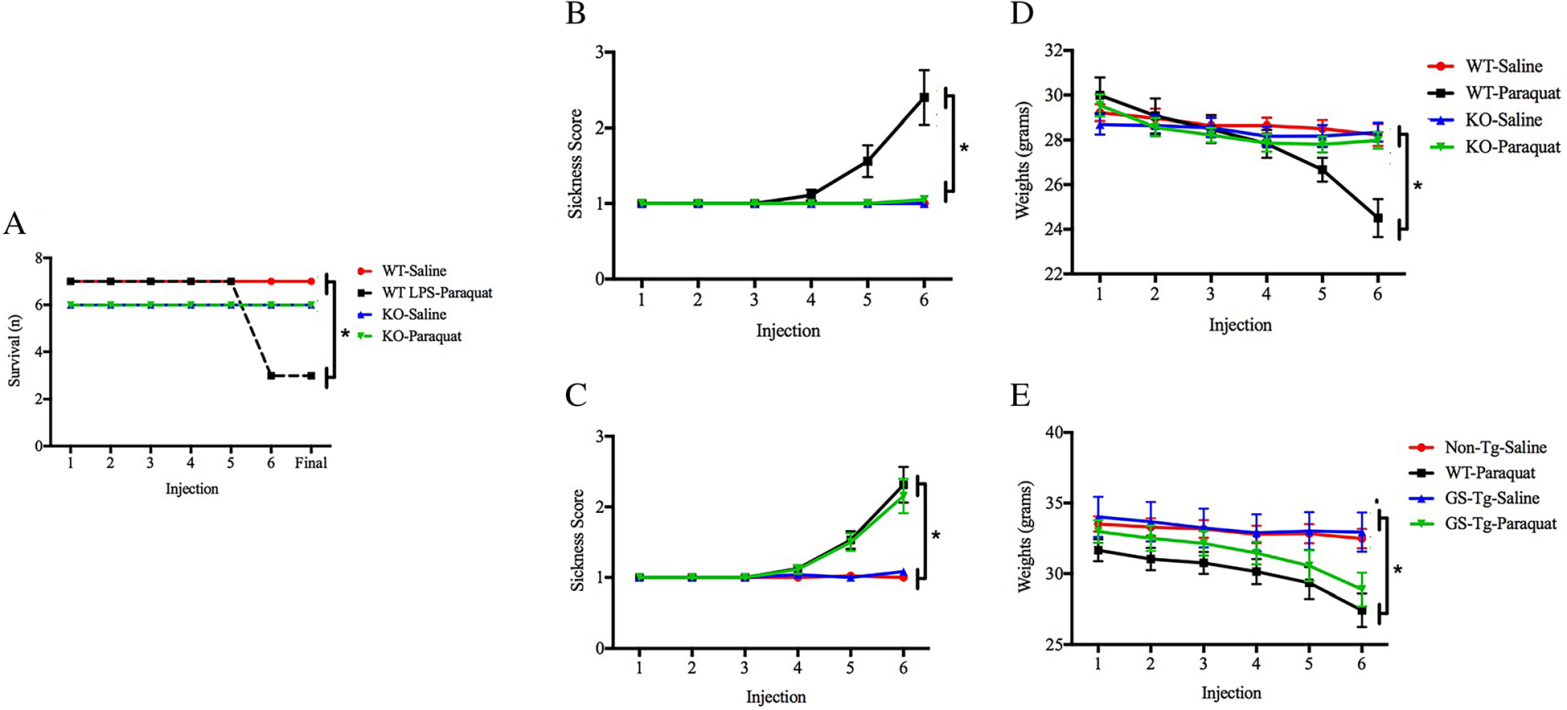
An initial study that involved intra-SNc LPS (2 μg) infusion followed by paraquat administration (10 mg/kg; ip) revealed that wild-type (WT) mice displayed signs of extreme sickness, with a significant number (6/9) of mice either died outright or had to be euthanized (owing to moribund presentation) by the ninth injection. In contrast, the mortality rate was reduced in LRRK2 knockout (KO) mice, such that only 1/6 reached end state following the LPS + paraquat regimen. (**a**) The follow-up study showed that in the absence of LPS and with a reduced paraquat injection regimen, paraquat-treated WT mice still displayed significant (albeit less) pathological signs in terms of ratings of sickness (**b**, **c**) and weight loss (**d**, **e**) by their sixth paraquat injection. Importantly, once again this effect was prevented by LRRK2 KO (**b**, **d**). However, the G2019S (GS-Tg) transgenic mice did not significantly differ from WT animals in terms of either sickness (**c**) or weight loss (**e**). Sickness was calculated as a composite score by blind raters including ptosis, piloerection, lethargy, and curled body posture. *p < 0.05, relative to respective controls

A preliminary study showed that by the ninth paraquat injection, a significant degree of mortality was observed in 6–8-month-old WT but not LRRK2 null mice (Fig. 1a). Indeed, significantly more WT mice that received paraquat treatment became moribund and hence reached endpoint compared to LRRK2 KOs or saline-treated mice (*p* < 0.05). This striking finding suggested that LRRK2 might have a fundamental role in processes aligned with inflammatory toxicity and hence, we reduced our paraquat injection regimen from nine to six injections. This allowed for better survival and determination of the impact of LRRK2 with regard to behavioral and systemic outcomes normally associated with immune and stress challenges. To this end, LRRK2 deficient and WT littermates between 6 and 8 months of age received paraquat (10 mg/kg; ip) or vehicle injection twice a week for 3 weeks. Moreover, a parallel study using 6–8-month-old transgenic mice overexpressing LRRK2 G2019S (which displays enhanced LRRK2 kinase activity) was also conducted using an identical paraquat injection regimen.

In agreement with the mortality finding, LRRK2 KO also prevented paraquat-induced signs of illness. Specifically, the repeated measures two-way ANOVAs revealed a genotype × injection interaction for scores of sickness (*F*(4,128) = 10.642, *p* < 0.001) and animal weights (*F*(5,160) = 8.319, *p* < 0.001) (Fig. 1b, d). Follow-up analyses revealed that beginning 1 day after the fifth injection, WT mice treated with paraquat had significantly lower weight and displayed higher sickness scores, relative to their saline-exposed littermates (*p* < 0.05), as well as with respect to LRRK2 null mice exposed to the toxin (*p* < 0.05). Correspondingly, the paraquat treatment promoted significant differences in weight (*F*(1,57) = 21.482, *p* < 0.001) and sickness ratings (*F*(1,52) = 13.05, *p* < 0.05) between the groups in the parallel G2019S study (Fig. 1c, e). However, in this case, the paraquat-induced signs of illness were not affected by the G2019S genotype. Indeed, the G2019S mutant mice appeared identical to their WT counterparts in their sickness response to paraquat. These findings suggest a role for endogenous LRRK2 in basic processes aligned with the development of sickness but that enhancement of its kinase activity does not add to such pathology.

### LRRK2 KO prevented paraquat-induced home-cage motor activity and nestlet building deficits

**Fig. 2:**
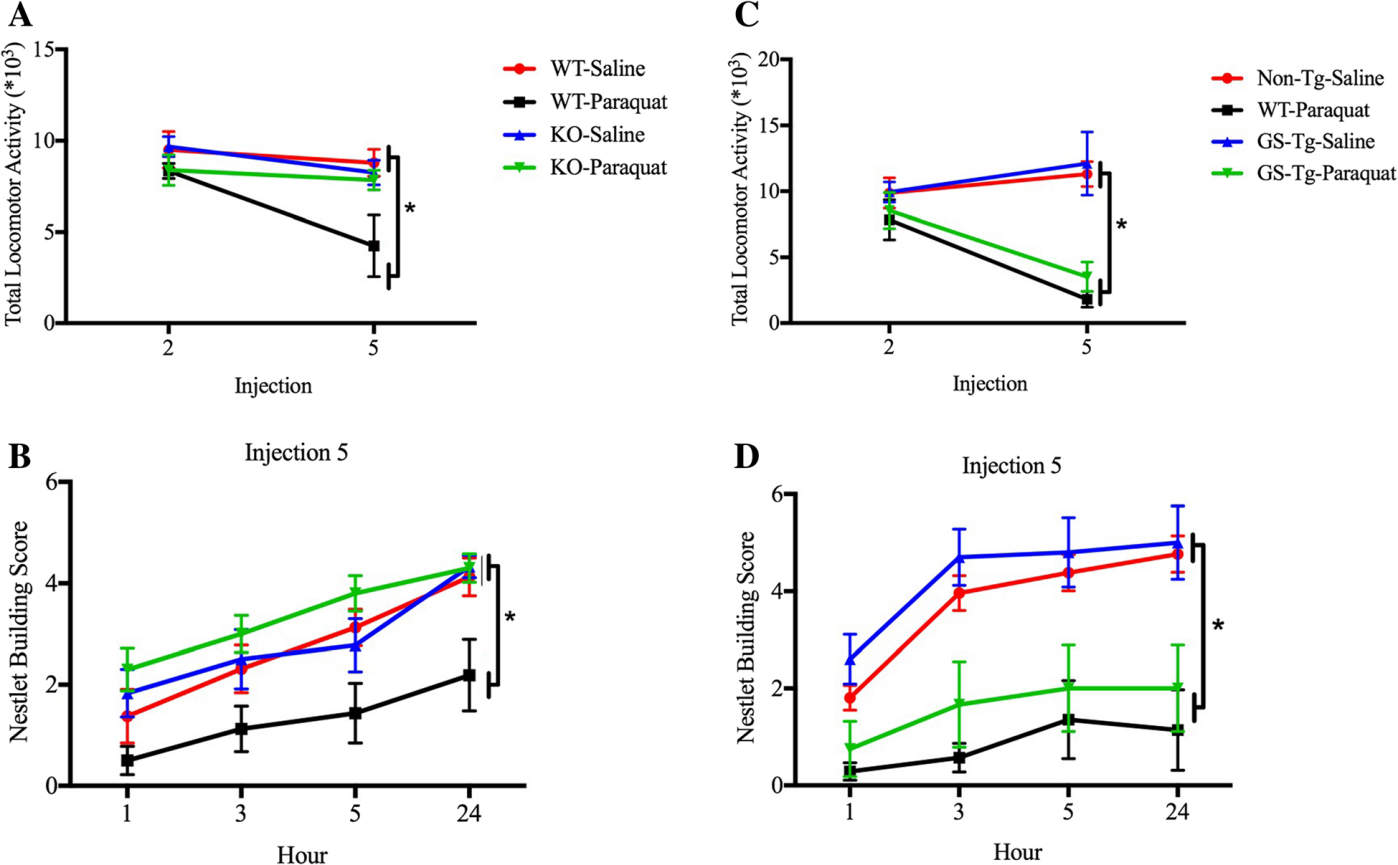
Wild-type (WT) mice displayed a significant reduction in home-cage activity by the fifth paraquat injection (10 mg/kg; ip), and this effect was reversed by LRRK2 knockout (KO) (**a**) but unaffected by G2019S (GS-Tg) transgenic overexpression (**c**). As a measure of nesting behavior, which requires fine motor skills and is influenced by stress, nestlet building was assessed in the 24 h following the fifth paraquat injection. Paraquat treatment markedly reduced nest building in WT mice, whereas LRRK2 KO prevented this deficit (**b**) but was unaffected by G2019S mutation (**d**). *p < 0.05, relative to respective controls

A genotype × injection interaction was observed for home-cage motor activity after the fifth paraquat injection (*F*(3,25) = 3.196, *p <* 0.05). Indeed, paraquat-treated WT mice displayed significantly less home-cage activity over a 24-h period (*p* < 0.05), but LRRK2 KO prevented this motor reduction (Fig. 2a). We also assessed nestlet (cage material used to construct a nest) building behavior, as this requires fine motor skills and is also linked to motivational processes that are typically disrupted by stressors. The repeated measures two-way ANOVA revealed a genotype × injection interaction (*F*(3,31) = 4.846; *p* < 0.05). As borne out by the follow-up comparisons, by the fifth injection, WT paraquat-treated mice had significantly lower nestlet building scores relative to all other groups (*p* < 0.05; Fig. 2b). Once again, the parallel G2019S experiment confirmed the paraquat-induced reduction in home-cage activity (*F*(1,58) = 7.60, *p* < 0.05) and nestlet building score (*F*(1,108) = 26.41, *p* < 0.05) in WT mice but found that the transgenic G2019S mutation had no influence on these parameters (Fig. 2c, d).

### LRRK2 KO blunted corticosterone elevations but not BDNF or 5-HT1A changes in paraquat-exposed mice

**Fig. 3:**
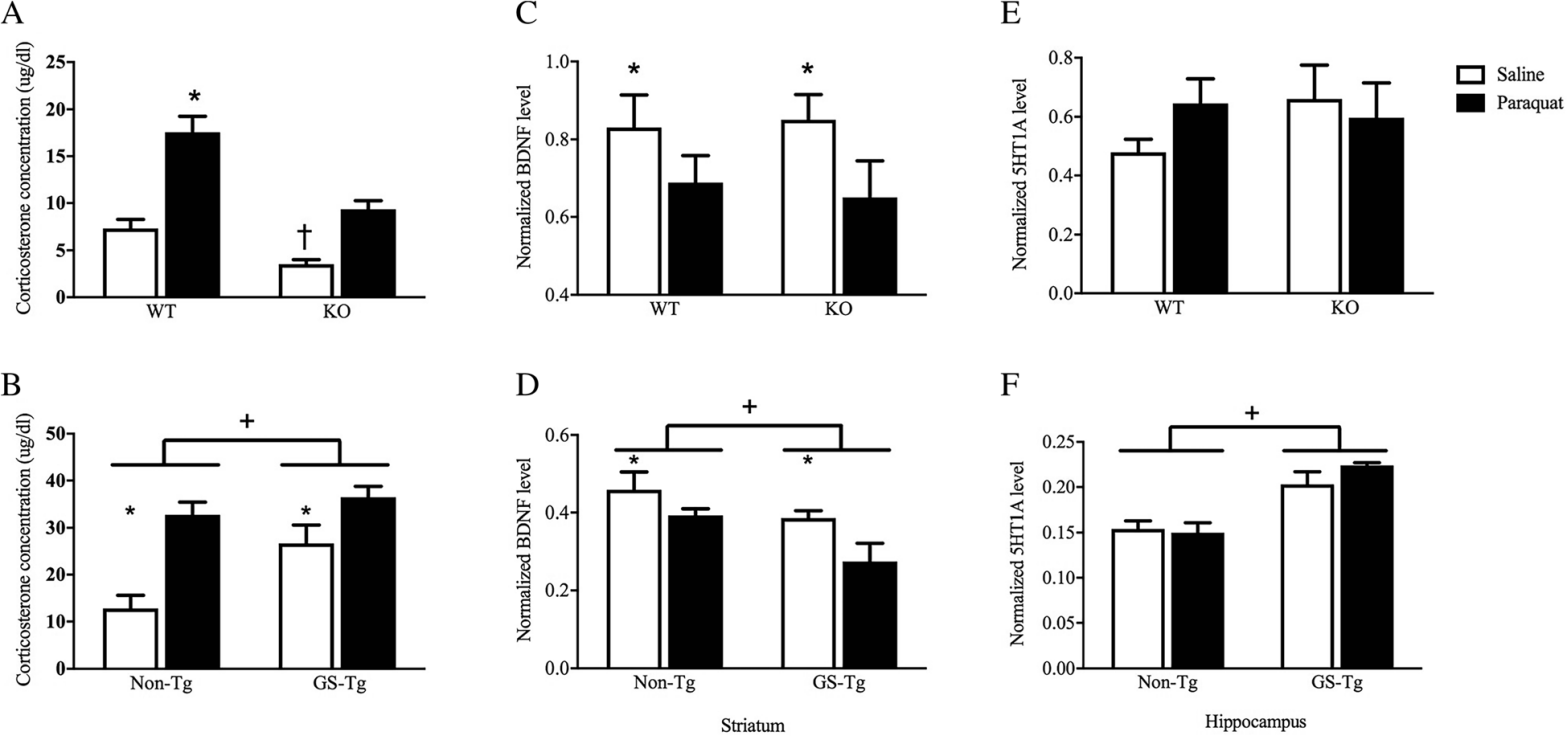
The paraquat treatment regimen (black bars) induced an elevation of plasma corticosterone levels, relative to saline treatment (white bars), and this elevation was significantly blunted in LRRK2 knockout (KO) mice (**a**). The G2019S (GS-Tg) transgenic mutation alone induced a significant corticosterone elevation that was similar to that induced by paraquat in non-transgenic mice (**b**). In both the LRRK2 KO and G2019S studies, paraquat significantly reduced striatal levels of BDNF in the absence of any effect of genotype (**c**, **d**). While hippocampal 5-HT1A levels were not affected by paraquat or LRRK2 KO (**e**), the G2019S mutation did elevate 5-HTIA, relative to saline treatment (**f**). *p < 0.05, relative to respective saline-treated controls, ^+^*p* < 0.05, difference between genotypes

Plasma corticosterone was assessed as an index of general “stress state” of mice and is also a useful measure that usually correlates with inflammatory sickness profiles. Once again, a significant genotype × injection interaction was evident (*F*(1,32) = 3.883, *p* = 0.05). Paralleling the sickness syndrome, Fig. 3 shows that paraquat clearly had a stressor-like effect as reflected by markedly elevated plasma corticosterone levels in WT mice, relative to saline injection; however, this effect was dramatically blunted in the LRRK2 KOs (*p* < 0.05; Fig. 3a). The transgenic G2019S study confirmed a significant main effect of paraquat (*F*(1,20) = 8.67, *p* < 0.05) and genotype (*F*(1,20) = 24.44, *p* < 0.05) on plasma corticosterone (Fig. 3b). Indeed, as shown in Fig. 3a, b, paraquat once again significantly increased corticosterone levels (*p* < 0.05), but most strikingly the G2019S overexpressing transgenic mice displayed further elevated levels of the stress hormone. In fact, G2019S mice even had significantly elevated basal levels of corticosterone, relative to their WT counterparts (*p* < 0.05).

In addition to the stressor-like effects of paraquat on corticosterone, similar alterations were observed with regard to levels of the trophic factor, brain-derived neurotrophic factor (BDNF), within the striatum (Fig. 3c, d). Indeed, striatal levels of BDNF were found to be reduced by paraquat treatment in both the LRRK2 KO (*F*(1,22) = 4.76, *p* < 0.05) and G2019S (*F*(1,15) = 5.81, *p* < 0.05) studies. Moreover, G2019S mice had basally reduced striatal BDNF levels (*F*(1,15) = 6.72, *p* < 0.05). However, LRRK2 deficiency did not further impact levels of the growth factor, but the transgenic G2019S mutation did significantly further reduce BDNF within the striatum, relative to their WT counterparts (*p* < 0.05). In contrast to the striatum, no significant changes in SNc levels of BDNF were evident in either of the studies.

As a further measure of the potential role of LRRK2 on stress-related processes, we assessed 5-HT1A levels in the hippocampus, which has been implicated in depression and anxiety that accompany PD [35]. To this end, although neither paraquat nor LRRK2 KO affected hippocampal 5-HT1A levels of the receptor were altered by the G2019S mutation (*F*(1,15) = 30.03, *p* < 0.05; Fig. 3e, f). Specifically, as shown in Fig. 3f, basal 5-HT1A levels were significantly increased in the G2019S transgenic mutants, relative to their WT littermates (*p* < 0.05).

**Fig. 4:**
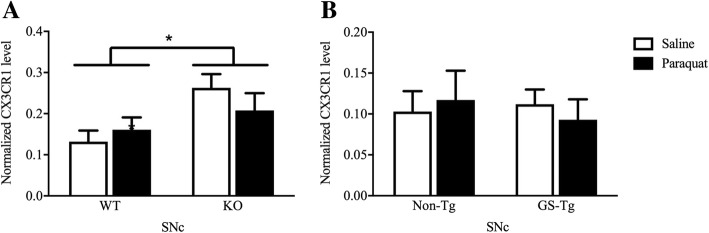
Substantia nigra (SNc) levels of the chemokine receptor, CX3CR1, which is selectively found on microglia were significantly increased in LRRK2 knockout (KO) mice (**a**), but were unaffected by the G2019S (GS-Tg) transgenic mutation (**b**). In no case did paraquat administration affect SNc levels of CX3CR1. *p < 0.05, difference between genotypes

Finally, the chemokine receptor, CX3CR1, was assessed as an index of inflammatory cell changes. Indeed, engagement of the CX3CR1 receptor on microglia by its ligand, fractalkine, has been reported to maintain a basal microglial phenotype and, in fact, might act in an anti-inflammatory capacity [40]. To this end, SNc levels of the chemokine receptor, CX3CR1 (which is found on microglia and infiltrating macrophages), did vary as a function of genotype (*F*(1,17) = 6.99, *p* < 0.05; Fig. 4a). Although paraquat had no effect on CX3CR1, levels of the inflammatory regulator were basally elevated by LRRK2 KO (*p* < 0.05; Fig. 4a). Hence, endogenous LRRK2 may act to restrict fractalkine signaling. In contrast, G2019S had no significant influence on SNc CX3CR1 levels (Fig. 4b).

### LRRK2 KO prevented the pathological effects of paraquat within the periphery

**Fig. 5:**
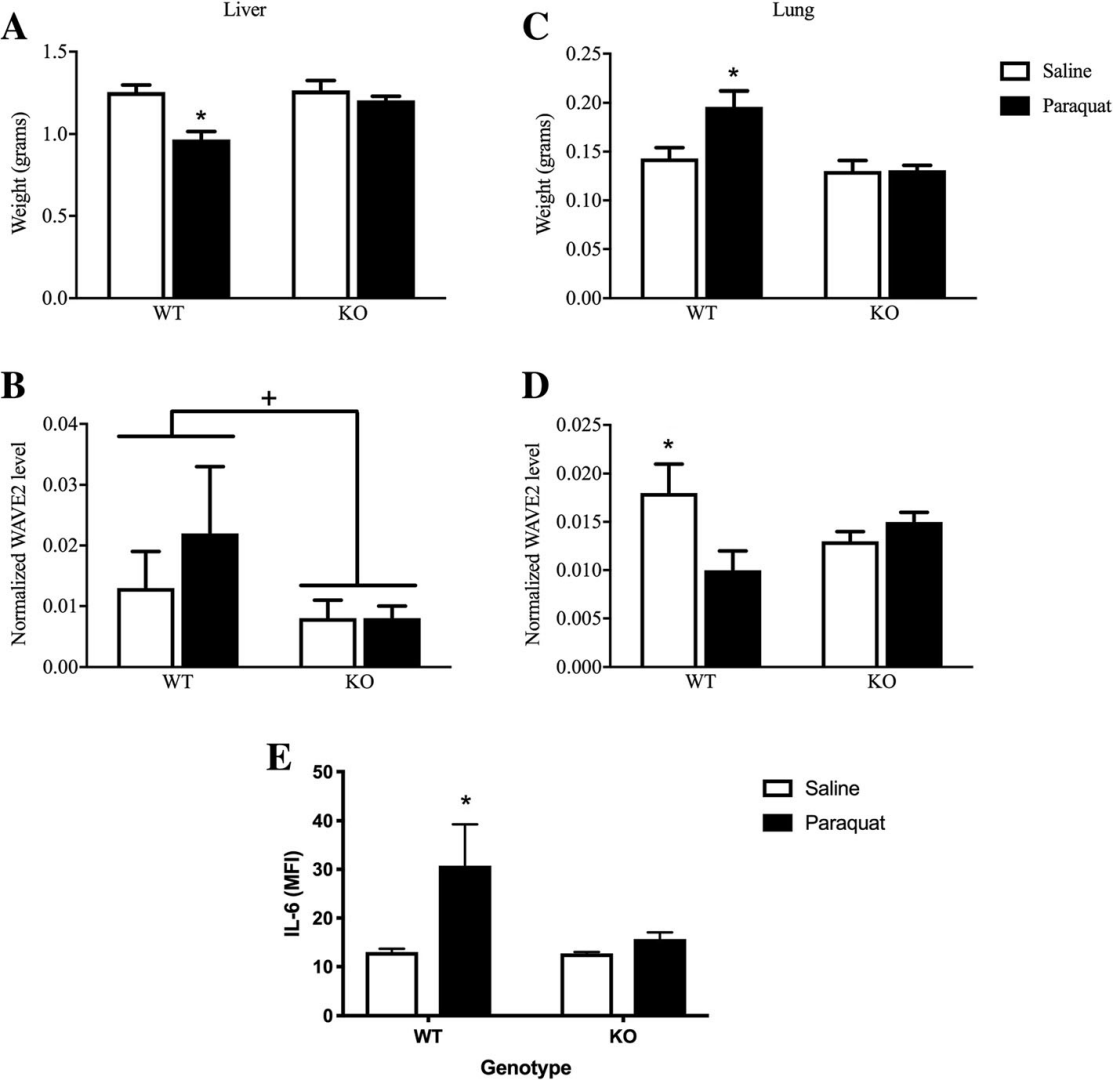
Assessment of gross organ weight revealed that paraquat reduced liver weight (**a**), while increasing lung weight (**c**) in wild-type (WT) mice, but these changes were completely absent in LRRK2 knockout (KO) mice. Levels of the inflammatory actin regulator protein, WAVE2, were reduced in the liver of LRRK2 null mice, relative to WT animals (**b**). Whereas WAVE2 levels in the lungs were reduced by paraquat in WT mice, no such effect was apparent in LRRK2 KOs (**d**). Finally, paraquat provokes a significant elevation of plasma IL-6 in WT mice, but LRRK2 KO prevented this effect (**e**). *p < 0.05, relative to respective saline-treated controls; ^+^*p* < 0.05 differences between genotypes

An additional study was conducted to assess peripheral organ changes, and this experiment was restricted to LRRK2 KOs, given that G2019S had few effects on parameters of paraquat toxicity. This study sought to assess whether the sickness changes induced were associated with changes in the periphery, namely, circulating IL-6 and changes in organ weight (indicative of atrophy) and inflammatory factor expression (WAVE2 and CX3CR1; indicative of inflammatory cell migration and potential tissue remodeling). Accordingly, paraquat injection parameters identical to the aforementioned studies were utilized in 6–8-month-old mice. Moving from the brain into the periphery, we took gross measures of organ weight of the liver, lungs, and spleen. These yielded significant genotype × injection interactions for the liver (*F*(1,32) = 6.381, *p* < 0.05; Fig. 5a) and lungs (F(1,32) = 4.369, *p* < 0.05; Fig. 5c). In the liver, paraquat reduced organ weight in WT animals (*p* < 0.05; Fig. 5a) and LRRK2 KO prevented this effect. In the lung, paraquat increased organ weight (*p* < 0.05) and again, this effect was prevented by LRRK2 ablation (Fig. 5c). The former effect could be related to liver atrophy, whereas the latter effect could be attributable to LRRK2 preventing the expected infiltration of immune cells and pneumatic inflammation that characterizes paraquat’s typical lung toxicity. No significant differences were observed for the spleen.

Surprisingly, in no case did organ levels of CX3CR1 vary as a function of the treatments (data not shown). Yet interestingly, levels of the actin regulatory factor, WAVE2, which is crucial for immune cell migration and phagocytotic responses did vary within the lungs as function of a genotype × injection interaction (*F*(1,12) = 5.752, *p* < 0.05; Fig. 5d). In this case, paraquat reduced WAVE2 levels in the lungs of WT mice but had no effect in the LRRK2 KOs, which already has basally suppressed levels of WAVE2 (*p* < 0.05). Within the liver, paraquat modestly (albeit not significantly) raised WAVE2 levels; but again, a dramatic effect was evident as a function genotype (*F*(1,12) = 4.703, *p* < 0.05; Fig. 5b), such that LRRK2 null mice displayed lower levels relative to their WT counterparts (*p* < 0.05; Fig. 5b). No significant differences were noted within the spleen, indicating some degree of specificity in the paraquat and LRRK2 KO effects at the organ level.

Finally, we assessed plasma cytokine levels and found significant variations in IL-6 as a function of a genotype × injection interaction (*F*(1,29) = 5.267, *p* < 0.05). The follow-up comparisons confirmed that circulating IL-6 was significantly elevated in paraquat-treated WT mice (*p* < 0.05), but that this effect was completely absent in the LRRK2 null mice (Fig. 5e).

## Discussion

LRRK2 is a large and complex gene that has been implicated in not only PD, but also other diseases that have an inflammatory component [41, 42]. It is thought to have fundamental immune functions, such as the regulation of monocyte and lymphocyte trafficking [9, 10], and a couple of reports indicate that LRRK2 is important for the neurodegenerative effects of toxicants, such as MPTP and rotenone [12, 25]. Yet, the role of LRRK2 in the context of paraquat exposure has not been explored, nor is it known how the gene might affect general inflammatory toxicity and stress processes. This is an important overlooked issue since PD, and in fact, virtually all neurological conditions are typically associated with marked stress and behavioral comorbidity and the same environmental toxins that have been linked to PD also cause inflammatory toxicity. In fact, we are interested in not only PD-like motor effects but also general sickness responses and the possibility that LRRK2 could be a mediator of fundamental body reactions to environmental stressors, particularly those that elicit oxidative and inflammatory distress, as paraquat does.

Our previous work showed that 2–3-month-old mice treated with paraquat displayed modest sickness behaviors which resolved rapidly, but these mice went on to display PD-like motor impairments [19, 34]. However, a small subset (~ 10–15%) of mice often does not fully recover and, hence, is removed from the experiment. In the present study, using moderately older mice (6–8 months), we found a much greater (~ 70%) mortality rate which became apparent after the sixth paraquat injection. Markedly, LRRK2 null mice were protected from this enhanced mortality, raising the possibility that the gene is involved in general toxicity. Hence, we reduced the number of paraquat injections with an aim of assessing whether LRRK2 is critical for the systemic stress and inflammatory toxicity of paraquat in older animals.

Consistent with our previous findings [43], paraquat acted as a systemic stressor, as evidenced by its elevation of plasma corticosterone and IL-6, provocation of sickness behavior, and disturbances in nesting and motor behaviors. Indeed, we and others have shown that paraquat-induced increased pro-inflammatory cytokine levels in the brain, as well as in the lungs and in serum [19, 32, 44–46]. We know that collectively these cytokines and corticosterone can act additively or even synergistically to provoke behavioral disturbances and neurochemical alterations and at high enough concentration can cause cellular death [47, 48]. Accordingly, paraquat presently induced peripheral changes indicative of inflammatory pathology. Just as was the case for the mortality, LRRK2 KO prevented these outcomes. Hence, indicating for the first time LRRK2 involvement in toxicant-induced sickness and stress factor responses and raising the possibility that it might influence brain circuits that are crucial for prototypical illness (e.g., shivering, fever, piloerection, ptosis) and stressor-relevant social species-specific behaviors [49, 50]. In effect, LRRK2 might be an important mediator of general inflammatory tone and brain-immune dialogue in the face of environmental insults.

Bi-directional communication between peripheral organs and the brain involving immune factors, such as cytokines or through neural fibers, has been well established. Indeed, cytokines can activate brain regions through vagal afferents or infiltration of the parenchyma through saturable transport mechanisms [51, 52]. For instance, nigral-vagal communication, which has been implicated in the gastrointestinal alterations in PD patients, may serve as a route for the spread of pathology, such as α-synuclein aggregates [53, 54]. In fact, direct stimulation of substantia nigra neurons was shown to activate the dorsal vagal complex, which in turn, regulated gastrointestinal functioning, and this communication was disrupted by paraquat administration [55]. As well, LRRK2 could play a role in gastric pathology in PD patients given that the LRRK2 MUC19 mutation confers risk of Chron’s disease [56] and that LRRK2 R1411G mutant mice display early gastrointestinal disturbances [57].

Besides the brain, the main target organ for paraquat is the lung, with the toxicant causing considerable pulmonary fibrosis and accumulation of inflammatory immune infiltrates [46, 58]. The increased lung weight presently observed with paraquat treatment is consistent with the edema and accumulation of immune cell infiltrates that has previously been reported with paraquat intoxication [59]. Moreover, the reduction in WAVE2 lung levels we observed might reflect general damage to tissue, or alternatively, variations in cell trafficking. WAVE2 is known to be crucial for the actin re-modelling in immune cells that are required for trafficking and processes such as phagocytosis [36, 60]. Indeed, LRRK2 was reported to control phagocytic immune cell responses through the actin regulatory factor, WAVE2 [36], which also may be fundamental for basic inflammatory cell trafficking and inflammatory phenotype [60, 61]. The fact that LRRK2 knockout mice had lower overall basal organ levels of WAVE2 is consistent with a role for LRRK2 in regulating WAVE2-dependent inflammatory processes [36, 60]. Specifically, WAVE2 would be expected to underlie phenotypic shifts in the activation state of inflammatory cells (particularly macrophages) and if this is regulated by LRRK2, then its deficiency would limit inflammatory cell migration into organs. Yet, the reduction in lung WAVE2 induced by paraquat in wild-type mice is hard to reconcile; it conceivably could reflect an increased migration of inflammatory immune cells from out of the lung into the circulatory or lymphatic system. Alternatively, alveolar macrophages are particularly vulnerable to paraquat toxicity and the reduction in WAVE2 could actually reflect a loss of these cells [62]. Interestingly, levels of the fractalkine receptor, CX3CR1, were not affected in the lung or other peripheral tissues but were basally elevated in the SNc of LRRK2 KOs. Emerging evidence suggests that CX3CR1 can act as a distress signal that mobilizes immune cells [63], as well as a regulator of basal immune cell housekeeping functions [64–66]. Our findings suggest clearly contrasting roles for WAVE2 and fractalkine and are consistent with the notion that the former is aligned with pro-inflammatory responses, and latter may foster more of an anti-inflammatory or “normal” homeostatic phenotype for microglia and peripheral immune cells.

Brain-lung interactions readily occur in the context of inflammatory pathology. For instance, direct inflammation of the lung via monocrotaline (used to induce pulmonary hypertension) causes microglial activation within the brain and this effect was reversed by anti-inflammatory (minocycline) treatment [67]. Similarly, brain pathology induced by traumatic brain injury provoked lung pathology characterized by neutrophil infiltration, alveolar thickening, and fibrin deposition [68]. Also, in addition to the lung, we found that paraquat-reduced liver weight, which is consistent with organ atrophy from repeatedly dealing with detoxification and excretion of the toxicant. Of course, an important caveat of this study is that organ histology was not performed, so it is unclear as to the extent to which paraquat might have directly damaged the tissues.

It was particularly interesting that the G2019S transgenic mutation did not affect overall toxicity, but that these mice did show signs of enhanced basal stress tone. Specifically, corticosterone levels and hippocampal 5-HT1A were basally increased, and also simply injecting the saline vehicle reduced home-cage activity levels in transgenic G2019S animals (compared to their WT littermates). The fact that the LRRK2 deficiency reduced the corticoid response, but the G2019S mutation increased it suggests a clear divergence in the role of endogenous LRRK2 vs. it is the increased kinase activity that occurs with G2019S mutation. LRRK2 is a complex protein with kinases activity, as well as non-kinase functions that involve its facilitation of protein-protein interactions. The G2019S mutation increases kinase activity but should not affect non-kinase mechanisms, suggesting a possible selective role for kinase-mediated actions in promoting corticoid responses (possibly by phosphorylating CRH or by stimulating p38 or JNK). Alternatively, knockout of endogenous LRRK2 clearly blunted the HPA response and this could stem from reduced kinase activity, as well as potential reductions in protein signaling pathways, most notably, p38, JNK, interferon gamma, as well as a number of RAB proteins that control vesicular trafficking, autophagy, and lysosomal functioning [69]. We and others did find that G2019S overexpression markedly altered basal and immune challenge evoked dopaminergic activity within the striatum [70–72]. But again, these transgenic mice did not show changes in pathology or inflammatory parameters, including sickness behaviors or circulating cytokines. Hence, forced G2019S overexpression clearly has functional effects but not necessarily those that would be obviously aligned with inflammatory damage or neurodegeneration.

The stress phenotype of the G2019S mice is intriguing in that it raises the possibility that the mutation could specifically favor the development of psychiatric disturbances, which are commonly co-morbid with PD [72–74]. Indeed, the basal elevation of 5-HT1A we observed in G2019S mutants is consistent with a recent report that also found these mice to display anxiety and depressive-like behaviors [35]. The LRRK2 BAC G2019S mice also showed decreased dopamine release [70], which could certainly contribute to behavioral disturbances. Similarly, the basally increased corticosterone we observed in the G2019S mice could conceivably have been modulating behaviors. It is important to note that unlike these 6–8-month-old animals, we recently found that 2-month-old G2019S overexpressing mice had no such basal behavioral or corticoid differences [72], suggesting that age is critical for the manifestation of the “stress” phenotype.

There is a reason to believe that the G2019S mutation has a modulatory role with regard to neuroplasticity. For instance, it was reported that aged 8–9 months G2019S over-expressing mice had impaired plasticity of medium spiny striatal neurons [75]. The G2019S mutation was also linked to altered hippocampal LTD [76], along with diminished adult hippocampal neurogenesis and dendritic arborization and spine numbers in newly generated neurons [77]. Yet, although paraquat did reduce BDNF, the present results found that neither LRRK2 KO nor the G2019S mutation affected hippocampal or striatal levels of the growth factor.

It is important to underscore the differences between bacterial artificial chromosome (BAC) G2019S transgenic over-expressers (as presently used), and targeted G2019S knock-in mice. Of course, with BAC G2019S overexpression, mutant LRRK2 levels are artificially induced plus there is still some expression of WT LRRK2. Hence, interactions between the two could conceivably take place, and we are essentially looking at the combined effects of ~ 6–8-fold over-expression of the mutant G2019S transgene in the context of the normal expression of WT LRRK2. In contrast, the knock-in form of G2019S entirely replaces the WT form and elicits more “normal” levels of the gene.

## Conclusions

We found that 6–8-month-old mice had marked sickness and behavioral disturbances in response to paraquat and LRRK2 knockout totally prevented these effects. Although it is likely that immune cells and/or inflammatory factors played a role in these outcomes, the elevation of corticosterone may also be important since it is known to modulate inflammatory processes and recovery from sickness [78]. Of course, the fact that the paraquat-induced cytokine changes (IL-6) could actually be driving the corticosterone response makes it difficult to disentangle the individual effects of cytokines vs hormonal changes. Whatever the case, endogenous LRRK2 appears to be critically involved in paraquat provoked widespread pathology and is specifically modulating the downstream effects at multiple targets. Curiously, overexpression of the G2019S transgene did not affect paraquat toxicity but did appear to convey a “stress-like” phenotype, at least with regard to corticosterone, hippocampal 5-HT1A, and home cage activity. These data clearly suggest a novel role for LRRK2 in modulating general inflammatory and sickness profiles in response to a systemic environmental stressor. Not only is this important from a mechanistic prospective, but also clinically given that LRRK2 inhibitors are already being explored. Ultimately, endogenous LRRK2 appears to be important for processes aligned with toxicological threats and sickness responses, whereas the G2019S mutation may modulate basal stress state.

## Abbreviations

Bac: Bacterial artificial chromosome
BDNF: Brain derived neurotropic factor
KO: Knockout
LPS: Lipopolysaccharide
LRRK2: Leucine-rich repeat kinase-2
MMx: Micromax
PD: Parkinson’s disease
WT: Wild-type
